# L'hemorragie grave du peripartum en milieu de reanimation dans un centre universitaire tunisien de niveau 3: épidémiologie et facteurs de risque de mortalité maternelle

**DOI:** 10.11604/pamj.2015.21.277.6147

**Published:** 2015-08-11

**Authors:** Laidi Ben Nasr, Sofiene Ben Marzouk, Mehdi Kehila, Hamed Jabri, Saber Thamleoui, Hayen Maghrebi

**Affiliations:** 1 Service d'Anesthésie-Réanimation, Centre de Maternité et de Néonatologie de Tunis, Tunis, Tunisie; 2 Service C du Centre de Maternité et de Néonatologie de Tunis, Faculté de Médecine de Tunis, Université Tunis El Manar, Tunisie

**Keywords:** Mortalité maternelle, hémorragie du péripartum, réanimation, morbidité, maternal mortality, peripartum hemorrhage, resuscitation, morbidity

## Abstract

L'hémorragie grave du péripartum demeure une des causes principales de mortalité maternelle. L'objectif de notre étude était de décrire le profil épidémiologique des patientes qui ont été prises en charge en milieu de réanimation suite à une hémorragie grave du péripartum et de rechercher d’éventuels facteurs de risque de mortalité. Notre étude est rétrospective descriptive et analytique. Nous avons inclus tous les cas d'hémorragie du péripartum ayant séjourné en unité de réanimation obstétricale du centre de maternité et de néonatologie de Tunis (CMNT) au cours de la période allant de janvier 2010 à Décembre 2013. Nous avons recueilli les paramètres démographiques, obstétricaux, ceux relatifs à la prise en charge chirurgicale et réanimatoire, les scores de gravité SAPS obstétrical et APACHEII, ainsi que la morbi-mortalité. Au total nous avons colligé 322 cas sur quatre ans. La répartition annuelle des patientes ainsi que les caractéristiques démographiques et obstétricales étaient comparables dans leur globalité sur les quatre années. Les pratiques thérapeutiques étaient également comparables. Le taux global de mortalité par hémorragie dans notre unité était à 4,7%, avec un taux annuel de mortalité stable. L'analyse des facteurs de risque de mortalité par hémorragie en milieu de réanimation a montré une association statistiquement significative entre la survenue du décès et les facteurs suivants: recours aux catécholamines, survenue de sepsis, œdème pulmonaire aigu, coagulation intravasculaire disséminée, insuffisance rénale aigue avec recours à l'hémodialyse, SDRA ou TRALI, atteinte neurologique grave, défaillance multiviscérale et arrêt cardiaque récupéré.

## Introduction

L'hémorragie grave du péri-partum demeure une des causes principales de mortalité maternelle. La réanimation vise à préserver le pronostic vital tout en encadrant l'hémostase chirurgicale. Cependant la réanimation a sa propre morbi-mortalité qui vient s'ajouter à celle de l’évènement hémorragique. L'objectif de notre étude était de décrire le profil épidémiologique des patientes qui ont été prises en charge en milieu de réanimation suite à une hémorragie grave du péripartum et de rechercher d’éventuels facteurs de risque de mortalité.

## Méthodes

Notre étude est rétrospective descriptive et analytique. Elle a été menée au Centre de Maternité et de Néonatologie de Tunis (CMNT) qui est la plus grande maternité universitaire de niveau 3 en Tunisie. Le CMNT est doté d'un service de réanimation obstétricale, il draine les cas d'obstétrique les plus compliqués du pays et réalise 16000 à 17000 accouchements chaque année. Dans notre étude, nous avons inclus tous les cas d'hémorragie du péripartum ayant séjourné en unité de réanimation obstétricale au cours de la période allant du 1^er^ janvier 2010 au 31 Décembre 2013. Nous avons recueilli les paramètres démographiques, obstétricaux, ceux relatifs à la prise en charge chirurgicale et réanimatoire, les scores de gravité SAPS obstétrical et APACHEII, ainsi que les complications et les décès. Nous avons calculé des moyennes, des médianes et des pourcentages relatifs à chaque année. Nous avons utilisé les tests de Chi deux, de Fisher et le test T de Student pour analyser les données. La recherche des facteurs de risque de décès s'est faite sur l'effectif global des quatre années. L'analyse a été faite à l'aide du logiciel SPSS^17^. Le seuil de signification statistique a été fixé à 0,05.

## Résultats

Nous avons colligé 322 cas d'hémorragie grave du péripartum qui ont séjourné en unité de réanimation. Nous avons tenu compte des données manquantes lors de la description et de l'analyse de chaque paramètre (7 cas). La répartition des patientes sur les quatre années ainsi que les caractéristiques démographiques et obstétricales étaient comparables dans leur globalité sur les quatre années (Tableau 1). L'HPP complique plus souvent les accouchements par césarienne que les accouchements par les voies naturelles, dans un contexte de prématurité. La prise en charge de l'hémorragie a toujours fait appel à des moyens chirurgicaux (Tableau 2) et à la réanimation. La réanimation reposait toujours et essentiellement sur la transfusion de produits sanguins labiles à savoir les concentrés de globules rouges (CGR), les unités de plasma frais congelé (PFC), des concentrés standards de plaquettes (CSP). Elle reposait aussi sur l'administration de concentrés de fibrinogène, sur les produits procoagulants comme l'acide tranéxamique et le facteur VII activé recombinant. Le recours à la radiologie interventionnelle était rare et réservé aux échecs ou insuffisance de l'hémostase chirurgicale (Tableau 2). L'utilisation des catécholamines était nécessaire dans 19 à 30% des cas. La durée moyenne de séjour en réanimation allait de 2 à 3 jours. L’éventail des complications secondaires à l'hémorragie grave du péripartum et au séjour en réanimation était large, leurs fréquences sont restées globalement stables sur la durée de l’étude (Tableau 3) excepté celles de la coagulation intravasculaire disséminée (CIVD), du sepsis et de l'insuffisance rénale aigue. Le taux global de mortalité par hémorragie dans notre unité était à 4,7%, avec un taux annuel de mortalité stable (Tableau 3). L'analyse des facteurs de risque de mortalité par hémorragie en milieu de réanimation qui a été faite sur l'effectif des 322 cas colligés sur quatre ans a montré une association statistiquement significative entre la survenue du décès et les facteurs suivants: recours aux catécholamines, survenue de sepsis, œdème pulmonaire aigu, coagulation intravasculaire disséminée, insuffisance rénale aigue avec recours à l'hémodialyse, SDRA ou TRALI, atteinte neurologique grave, défaillance multiviscérale, et arrêt cardiaque récupéré (Tableau 4).

**Tableau 1:** Paramètres démographiques et obstétricaux

Année	2010	2011	2012	2013	p
Nombre	116	50	85	64	--
Age moyen (année)	31,7 ± 5,5	32,1 ± 5,7	31,9 ± 5,5	32,9 ± 5	0,26
Parité (médiane)	2 [1-8]	1 [1-5]	2 [1-5]	2 [0-8]	0,27
Gestité (médiane)	2 [1-7]	1 [1-5]	2 [1-10]	2 [1-7]	0,35
Terme moyen (semaine)	37 ± 2,9	35,9 ± 3,6	37,1 ± 3,3	35,6 ± 3,8	0,02
Statut ASA (%):					0,11
1	97 (83,6%)	35 (70,0%)	73 (85,9%)	46 (71,9%)	
2	19 (16,4%)	15 (30,0%)	11 (12,9%)	17 (26,6%)	
3	0	0	1 (1,2%)	1 (1,6%)	
Postpartum (%) Péripartum (%)	115 (99,1%) 1 (0,9%)	48 (96%) 2 (4%)	85 (100%) 0	63 (98,4%) 1 (1,6%)	0,34
AVB (%)	35 (30,2%)	13 (26,0%)	28 (32,9%)	18 (28,1%)	0,61
AVB+Forceps (%)	9 (7,8%)	1 (2,0%)	4 (4,7%)	1 (1,6%)	
Césarienne (%)	72 (62,1%)	36 (72,0%)	53 (62,4%)	45 (70,3%)	

**Tableau 2:** Geste chirurgical/reprises/Radiologie interventionnelle

Année	2010	2011	2012	2013
Triple Ligature vasculaire (%)	11(12,9%)	3 (8.8%)	4(6,8%)	5(11,9%)
Ligature artères Hypogastriques (%)	40(47,1%)	18 (52.9%)	14 (23,7%)	8(19,0%)
Hystérectomie (%)	34(40,0%)	12 (35.3%)	31(52,5%)	19(45,2%)
Capitonnage utérin seul (%)	0 (0%)	0 (0%)	1 (1,7%)	0 (0%)
Geste vasculaire +capitonnage (%)	0 (0%)	1 (2.9%)	9(15,3%)	10(23,8%)
Reprise Chirurgicale (%)	20 (30,8%)	9 (22,0%)	20 (23,8%)	14 (22,6%)
Radiologie interventionnelle (%)	3 (2,6%)	2 (4%)	5 (5,9%)	2 (3,2%)

**Tableau 3:** Morbidité, mortalité

Année	2010	2011	2012	2013	p
Œdème pulmonaire aigu (%)	20 (17,2%)	9 (18,0%)	5 (5,9%)	11 (17,2%)	0,9
Sepsis(%)	13 (11,2%)	5 (10,0%)	5 (5,9%)	5 (7,8%)	0,6
CIVD(%)	21 (18,1%)	19 (38%)	3 (3,5%)	11 (17,2%)	<10^-3^
Insuffisance rénale aigue(%)	12 (10,3%)	10 (20,0%)	3 (3,5%)	7 (10,9%)	0.05
Atteinte neurologique(%)	4 (3,4%	6 (12,0%)	2 (2,4%)	3 (4,7%)	0,9
Etat de choc(%)	16 (13,8%)	10 (20,0%)	10 (11,8%)	9 (14,1%)	0,7
Thrombopénie(%)	41 (35,3%)	31 (62,0%)	26 (30,6%)	33 (51,6%)	0,02
SDRA/ TRALI(%)	5 (4,3%)	4 (8%)	2 (2,4%)	1 (1,6%)	0,37
Complications thromboemboliques(%)	1 (0.9%)	2(4%)	3(3.5%)	2(3.1%)	0,66
Défaillance multiviscérale(%)	8(6.9%)	4(8%)	3(3.5%)	5(7.8%)	0,67
Arrêt cardiaque récupéré(%)	2(1,7%)	3(6%)	2(2,4%)	3(4%)	0,66
Mortalité annuelle	5(4,3%)	4(8,0%)	2(2,4%)	4(6,3%)	0,55

**Tableau 4:** Les facteurs de risque de décès

Facteurs de risque de décès	p
Transfusion massive	0,07
Catécholamines	<10^-3^
Hémodialyse	0,49
Sepsis	0,03
Œdème pulmonaire aigu	0,01
CIVD	<10^-3^
Insuffisance rénale aigue	<10^-3^
Atteinte neurologique	<10^-3^
Etat de choc	<10^-3^
SDRA /TRALI	<10^-3^
Complications thromboemboliques	0,05
Défaillance multiviscérale	<10^-3^
Arrêt cardiaque récupéré	<10^-3^

## Discussion

Le taux annuel de mortalité dans notre unité varie entre 2,4 et 6,3%. La morbidité associée à la prise en charge des HPP en milieu de réanimation n'est pas négligeable en termes d'incidence et d'impact sur la mortalité. L´hémorragie du post-partum (HPP) sévère est définie comme une hémorragie issue de la filière génitale excédant 1000 mL pour l´accouchement par voie basse et 1500 mL après une césarienne [1]. Afin d'optimiser la prise en charge de l'HPP, le Collège national des gynécologues et obstétriciens français (CNGOF) distingue dans ses recommandations 3 groupes d'HHP: les HPP répondant aux mesures obstétricales initiales, les HPP sévères requérant la mise en route du sulprostone et les HPP graves nécessitant le recours à une technique invasive d'hémostase [2]. Au sein du CMNT, l'hémorragie du péripartum grave représente le 1er motif d'admission en réanimation (44,9%) et la 2^ème^ cause de mortalité maternelle (28,1%) après le sepsis. Parmi les 61134 accouchements réalisés au CMNT, 322 se sont compliqués d'une HPP grave nécessitant l'hospitalisation en milieu de réanimation soit une incidence de 0,51%.

En France, entre décembre 2004 et novembre 2006, l'incidence de l'HPP grave était de 1,7% avec des extrêmes allant de 0% dans une maternité effectuant 125 accouchements par an, à 4% dans les maternités ayant une activité plus importante [3]. L'hémorragie du péripartum grave continue à être l´une des toutes premières causes de mortalité maternelle en France [4]. Dans notre étude, le taux global de mortalité secondaire à une hémorragie grave était de 4,7%. Les facteurs de risque de mortalité maternelle (MM) secondaire à l'HHP sévère classiquement décrits dans la littérature ont été retrouvés. Ce risque reste toujours corrélé à l´âge maternel [5]. Selon Bouvier et al, ce risque est minimum entre 20 et 24 ans et reste faible jusqu´à 29 ans. Il augmente très nettement à partir de 35 ans puisqu'il est trois fois plus élevé de 35 à 39 ans et douze fois plus élevé à 45 ans par rapport au groupe de 20 à 24 ans [6]. Dans la série de Fenomanana [7], l’âge supérieur à 35 ans représente 12,2 fois plus de risque de mortalité secondaire à une hémorragie du post-partum par rapport au groupe de 18 à 34 ans. Ceci s'explique par le fait que la multiparité est souvent associée à un âge avancé (>30 ans). Dans cette étude, 19 cas (55,88%) étaient multipares [7].

Dans notre série, 73% des patientes étaient multipares avec un âge moyen de 32 ans ± 5. Parmi l'ensemble des HPP identifiées, la proportion de cas graves était significativement plus importante pour les HPP après césarienne (65,4%) que pour les HPP après accouchement par voie basse 34,6%.

Afin de prédire la gravité et améliorer la prise en charge de L'HPP, Gayat et al [8], ont établi un score de gravité d'HPP incluant cinq facteurs prédictifs de gravité indiquant la nécessité de recours à un geste hémostatique à savoir Une anomalie de la placentation, une tachycardie supérieure à 115 bpm, un Taux de prothrombine inférieur à 50%, un taux de fibrinogène inférieur à 2 g/dL et un taux de troponine I détectable. Le taux de fibrinogène semble être un facteur indépendant prédictif de sévérité des HPP [9]. En effet, un taux inférieur à 2g/l aurait une valeur prédictive positive de 100%, alors qu'un taux supérieur à 4 g/l aurait une valeur prédictive négative de 79% [10]. Nous ne disposons pas de données suffisantes sur les taux sanguins de fibrinogène, pour pouvoir les inclure dans notre analyse. Peu d’études ont recherché les complications prédictives de mortalité maternelle au décours de la prise en charge l'HPP grave en milieu de réanimation. Dans notre série, une association statistiquement significative a été retrouvée entre la survenue du décès et la transfusion massive, le recours aux catécholamines, la survenue d'une insuffisance rénale aigue nécessitant l'hémodialyse, le sepsis, la CIVD, l’œdème pulmonaire aigu, la défaillance multiviscérale, l’état de choc et l'atteinte neurologique grave. Les facteurs de risque de mortalité maternelle par HPP rapportés dans la littérature [7] sont essentiellement: l’âge supérieur à 35 ans, la parité supérieure à 4, le travail long supérieur à 13 heures et le retard de prise en charge de plus d'une heure. Dans ce contexte, Il est toujours intéressant de noter que la clinique a encore toute sa place dans l’évaluation de la gravité de la situation. Différents facteurs d'ordre pratique ou logistique peuvent aboutir à l'aggravation de la situation avant l'arrivée en réanimation en particulier la mauvaise organisation, le manque de moyens humains et techniques.

Les limites de notre étude sont essentiellement son caractère rétrospectif et l'absence de groupe témoin. L'autre limite repose sur le fait que nos données correspondent à une gestion de deuxième et parfois de troisième ligne, puisque la prise en charge initiale a été effectuée dans plusieurs cas dans d'autres structures avant d'effectuer le transfert en unité de réanimation.

## Conclusion

L'HPP est la deuxième cause de mortalité maternelle en milieu de réanimation. Sa prévention est un objectif majeur étant donné sa prévalence et ses conséquences sur la morbidité et la mortalité maternelles. Cette prévention passe par l'identification des situations à risque et la mise en place d'une surveillance adaptée permettant un diagnostic et une prise en charge précoces et rapides. Une réanimation adaptée doit permettre de diminuer l'incidence de la morbi-mortalité maternelle par hémorragie.
